# Transcription regulation of iron carrier transport genes by ECF sigma factors through signaling from the cell surface into the cytoplasm

**DOI:** 10.1093/femsre/fuac010

**Published:** 2022-02-09

**Authors:** Volkmar Braun, Marcus D Hartmann, Klaus Hantke

**Affiliations:** Department of Protein Evolution, Max Planck Institute for Biology, Max Planck Ring 5, 72076 Tübingen, Germany; Department of Protein Evolution, Max Planck Institute for Biology, Max Planck Ring 5, 72076 Tübingen, Germany; IMIT Institute, University of Tübingen, Auf der Morgenstelle 28, 72076 Tübingen, Germany

**Keywords:** extracytoplasmic sigma factor, siderophore, ECF, iron, *Escherichia coli*, *Pseudomonas*

## Abstract

Bacteria are usually iron-deficient because the Fe^3+^ in their environment is insoluble or is incorporated into proteins. To overcome their natural iron limitation, bacteria have developed sophisticated iron transport and regulation systems. In gram-negative bacteria, these include iron carriers, such as citrate, siderophores, and heme, which when loaded with Fe^3+^ adsorb with high specificity and affinity to outer membrane proteins. Binding of the iron carriers to the cell surface elicits a signal that initiates transcription of iron carrier transport and synthesis genes, referred to as “cell surface signaling”. Transcriptional regulation is not coupled to transport. Outer membrane proteins with signaling functions contain an additional N-terminal domain that in the periplasm makes contact with an anti-sigma factor regulatory protein that extends from the outer membrane into the cytoplasm. Binding of the iron carriers to the outer membrane receptors elicits proteolysis of the anti-sigma factor by two different proteases, Prc in the periplasm, and RseP in the cytoplasmic membrane, inactivates the anti-sigma function or results in the generation of an N-terminal peptide of ∼50 residues with pro-sigma activity yielding an active extracytoplasmic function (ECF) sigma factor. Signal recognition and signal transmission into the cytoplasm is discussed herein.

## Teaser

The special features of the iron carrier transport gene regulation including signaling from the cell surface and ECF sigma factors is discussed.

## Introduction

Iron is essential for almost all organisms. It is contained in the active center of many redox enzymes and in abundant proteins such as hemoglobin, myoglobin, and hemopexin. In bacteria, Fe^2+^ is readily available under anoxic conditions and is taken up by the bacterial Feo transport system (Kammler *et al*. 1993). However, iron acquisition by aerobic bacteria is hampered by the insolubility of Fe^3+^ (∼10^–6^ mM) and the formation under oxic conditions of polymeric hydroxy-aquo complexes. Thus, to overcome iron limitation, bacteria have evolved Fe^3+^ carriers, referred to as siderophores. In fact, bacteria and fungi synthesize more than 500 different siderophores and use not only their own siderophores but also those from other species (Hider and Kong 2010).

Since a surplus of intracellular iron is toxic (Braun 1997), as it promotes the formation of oxygen radicals that destroy DNA, proteins, and membranes, bacteria have developed systems to regulate the cellular iron concentration, primarily by: (i) activation of the transcription of siderophore synthesis and transport genes by extracellular Fe^3+^siderophores and (ii) the repression of gene transcription by the Fur protein (Hantke 1981, 2001). Under iron-sufficient growth conditions, Fur is partly loaded with a [2Fe-2S] cluster (Fontenot *et al*. 2020) that may act as a sensor of the intracellular iron status and may repress transcription of the genes encoding iron supply systems. The DNA-binding properties of the cluster remain to be determined.

In the following, we review the transcriptional regulation of the transport systems of Fe^3+^ complexed by citrate in *E. coli*, various siderophores in different *Pseudomonas* species, and heme in *Serratia marcescens*. They have in common recognition and signal generation by specific outer membrane proteins, signal transmission across the outer membrane and the periplasm, proteolytic fragmentation of the sigma regulatory protein followed by the activation of the sigma factor. Transcription is controlled by extracytoplasmic function (ECF) sigma factors (Chevalier *et al*. 2019, Helmann 2002, Otero-Asman *et al*. 2019). This review is focused on transcription regulation through signaling of iron carrier transport genes by selected gram-negative bacteria and does not intend to cover microbial iron transport. Readers interested in a more general view on TonB-dependent outer membrane proteins (TDP) involved in transport of a variety of substrates and in cell surface signaling are referred to reviews containing extensive bioinformatic analyses (Koebnik 2005, Noinaj *et al*. 2010, Bolam and van den Berg 2018). Besides the basic structure of a β barrel forming a pore with a plug inside, TDPs may contain an additional signaling domain and an extra N-terminal extension with unknown function. They are particularly abundant in Bacteroidetes (Pollet *et al*. 2021) and in Sphingomonads (Samantarrai *et al*. 2020). The genome of *Bacteroides thetaiotaomicron* predicts 121TDPS, the genome of *Sphingomonas fuliginis* 75 TDPs.The genes encoding the predicted TDPs frequently contain common regulatory elements and coexist with genes associated with transport of the cognate substrates across the cytoplasmic membrane and their metabolism. All kinds of poly- and oligosaccharides, organic compounds including xenobiotics, metal ions, heme, and cobalamins serve as substrates. Only a few predicted systems were studied experimentally. The easiest access to identify substrates is growth of the bacteria in the presence of the predicted substrate and identification of the induced TDP by SDS-gelelectrophoresis.

## Recognition of the signaling molecule and transmission of the signal across the outer membrane

Because the concentration of Fe^3+^siderophores in the medium is very low (Raymond *et al*. 2015), diffusion would not satisfy the cellular iron demand and the complexes are too large, close to 1 kDa, to diffuse through the porins of the outer membrane (OM) into the periplasm (Nikaido 2003). Instead, Fe^3+^-loaded siderophores (Fe^3+^siderophores) are transported into bacterial cells by specific transport systems that begin with the adsorption of the Fe^3+^siderophores to OM proteins that bind them with high affinity and specificity (Braun 2013, Visca *et al*. 2002). Transport across the OM consumes energy that is provided by the electrochemical potential of the cytoplasmic membrane. Transport across the cytoplasmic membrane is achieved by Fe^3+^siderophore-specific ABC transporters. Within the cytoplasm, Fe^3+^ is released from the siderophores by reduction to Fe^2+^ and is incorporated into proteins, which thereby acquire redox properties.

### Structure and function of the outer membrane receptor proteins

**Figure 1.**
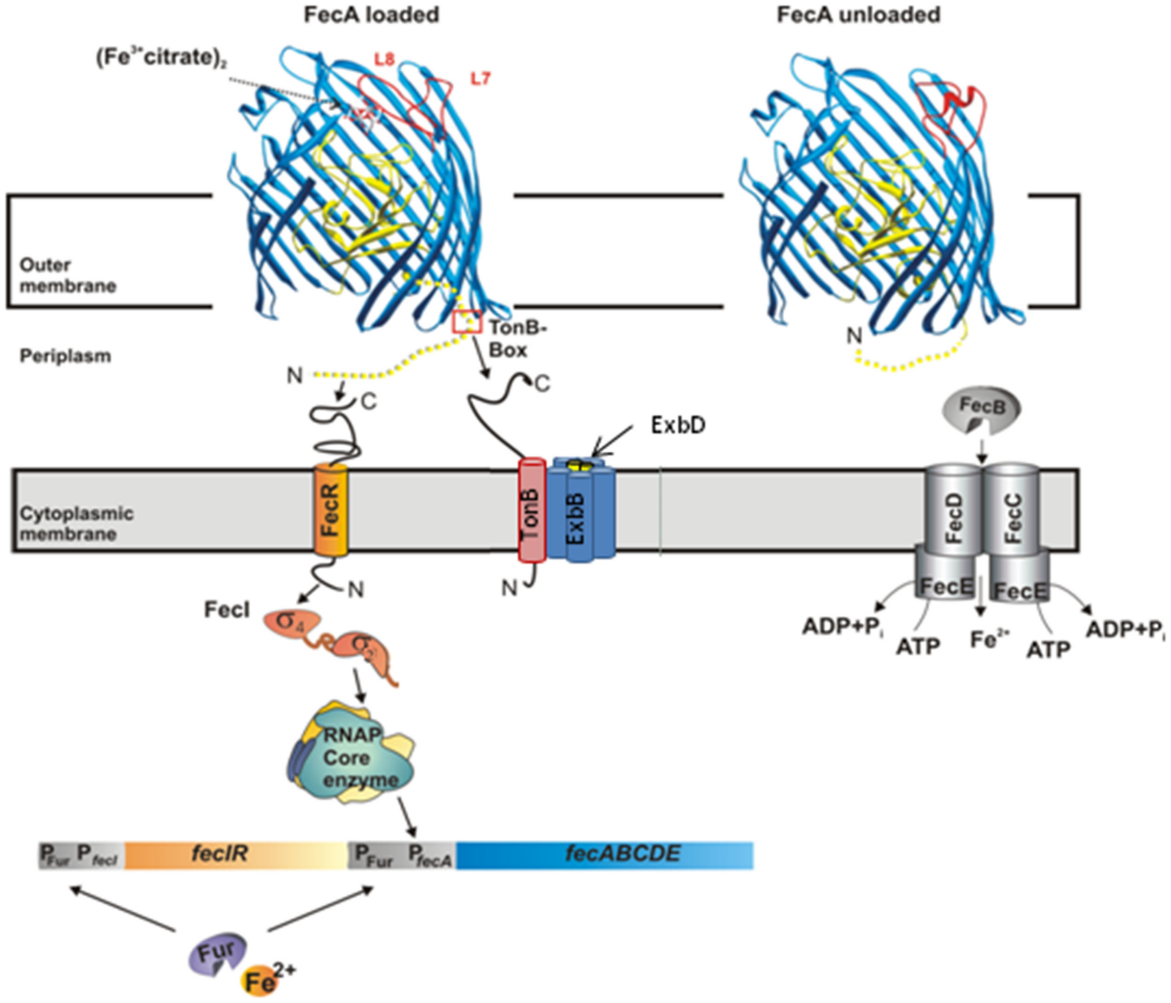
(Fe^3+^citrate)_2_ transport and regulatory system of *E. coli* K-12 The system includes the FecA regulatory and transport protein in the outer membrane, the FecR regulatory protein (pro-sigma factor) extending from the periplasm across the cytoplasmic membrane into the cytoplasm, the FecI sigma factor, and the FecB, FecC, FecD, and FecE proteins that form an ABC transporter in the cytoplasmic membrane. Binding of (Fe^3+^citrate)_2_ to FecA induces large conformational changes in FecA, in particular movements of the receptors L7 and L8 loops (compare FecA loaded with (Fe^3+^citrate)_2_ with unloaded FecA) which include the entire FecA structure up to the signaling domain at the periplasmic side. The signaling domain is not shown but is included in Figs 3 and 4. FecI with attached FecR directs RNA polymerase (RNAP core enzyme) to the *fecA* promoter, whereas σ^70^ controls *fecIR* transcription. The Fur protein, probably loaded with [2Fe-2S], represses the transcription of genes downstream of the *fecA* and *fecI* promoters. TonB binds to the TonB box of FecA. The complex formed by TonB, ExbB, and ExbD harvests the energy of the proton-motive force across the cytoplasmic membrane and transmits it to FecA.

The monomeric OM receptor proteins are approximately 80 kDa in size and consist of a 22-stranded β-barrel C-terminal domain that forms a pore that is occluded by the N-terminal plug domain (Fig. 1). A single OM protein may display an extraordinarily wide spectrum of activities: it may transport substrates and antibiotics structurally related and unrelated to those substrates, serve as a specific receptor that allows the binding of different phages and subsequent infection, take up bactericidal peptides and proteins, and act as signal receivers and signal transmitters. For example, the *E*. *coli* FhuA protein transports the fungal Fe^3+^siderophore ferrichrome, the structurally related antibiotic albomycin, the structurally unrelated microcin 25, and a synthetic rifamycin derivative across the OM membrane, imports colicin M, and serves as a binding site for the phages T1, φ80, T5, and UC-1 (Braun 2009).

In nearly all of these activities the OM proteins are not passive binding sites but must be energized by the proton-motive force of the cytoplasmic membrane, through the action of the Ton complex (Braun 1995). The latter consists of five copies of ExbB, two copies of ExbD, and TonB with an unknown stoichiometry (Celia *et al*. 2019). Ton complex proteins are inserted into the cytoplasmic membrane and extend into the periplasm (Kampfenkel and Braun 1992, Kampfenkel and Braun 1993). The region around residue 160 of TonB binds to the TonB box of TonB-dependent OM transporters. The TonB box consists of approximately seven residues, is located close to the N-terminus, and assumes a flexible conformation. Signaling OM proteins contain an N-terminal extension, designated the signaling domain that moves the TonB box towards the plug of the OM protein. Analyses of co-crystals of a C-proximal TonB fragment with the OM protein FhuA (Pawelek *et al*. 2006) and the vitamin B12 transporter BtuB (Shultis *et al*. 2006) showed that the TonB box forms a β-strand when it is attached to the C-proximal β-sheet of TonB, made up of three antiparallel β-strands. Cysteine cross-links introduced between TonB and the TonB box of FecA (the OM protein of the ferric citrate transport and signaling system, discussed below) and the properties of proteins carrying suppressor mutations demonstrate the *in vivo* binding of the two regions (Ogierman and Braun 2003). In contrast to unloaded FecA, the TonB box of FecA loaded with ferric citrate is not seen in the crystal structure, which indicates structural changes affecting its function. How the TonB complex reacts to the proton-motive force of the cytoplasmic membrane, harvests, and transmits the energy to OM proteins is unknown (Ratliff *et al*. 2021). One model proposes a rotary mechanism, similar to the proton-driven bacterial flagellar motor. The torque is generated by converting the energy of proton translocation through the transmembrane proton channel of the MotA/MotB complex (Santiveri *et al*. 2020). The MotB and ExbD sequences are homologous and the single transmembrane region of both proteins includes an Asp residue that is important for their activity (Braun *et al*. 1996). According to this scenario, ExbD together with ExbB couples the proton-motive force to TonB, which then transmits this energy to OM transporters (Ollis and Postle 2012).

Substrates of OM receptor proteins bind to loops at the cell surface and to residues of their plug domain. The large conformational change induced by substrate binding extends to the periplasmic domain but does not open the pore. Rather, the interaction of TonB with the TonB box of the OM protein converts the plug structure to reveal the pore, thus allowing passage of the ligand attached to the surface of the OM proteins into the periplasm.

### Signal recognition and signal transmission by the FecA outer membrane protein in response to ferric citrate (FeCit) in the medium

We begin description of the transcription regulation of iron carrier transport genes with discussion of the ferric citrate transport system. We will then discuss the regulation of the Fe^3+^sideriophore transport systems of Pseudomonas species and of the hemophore-mediated hemin transport system of *Serratia marcescens*.

**Figure 2.**
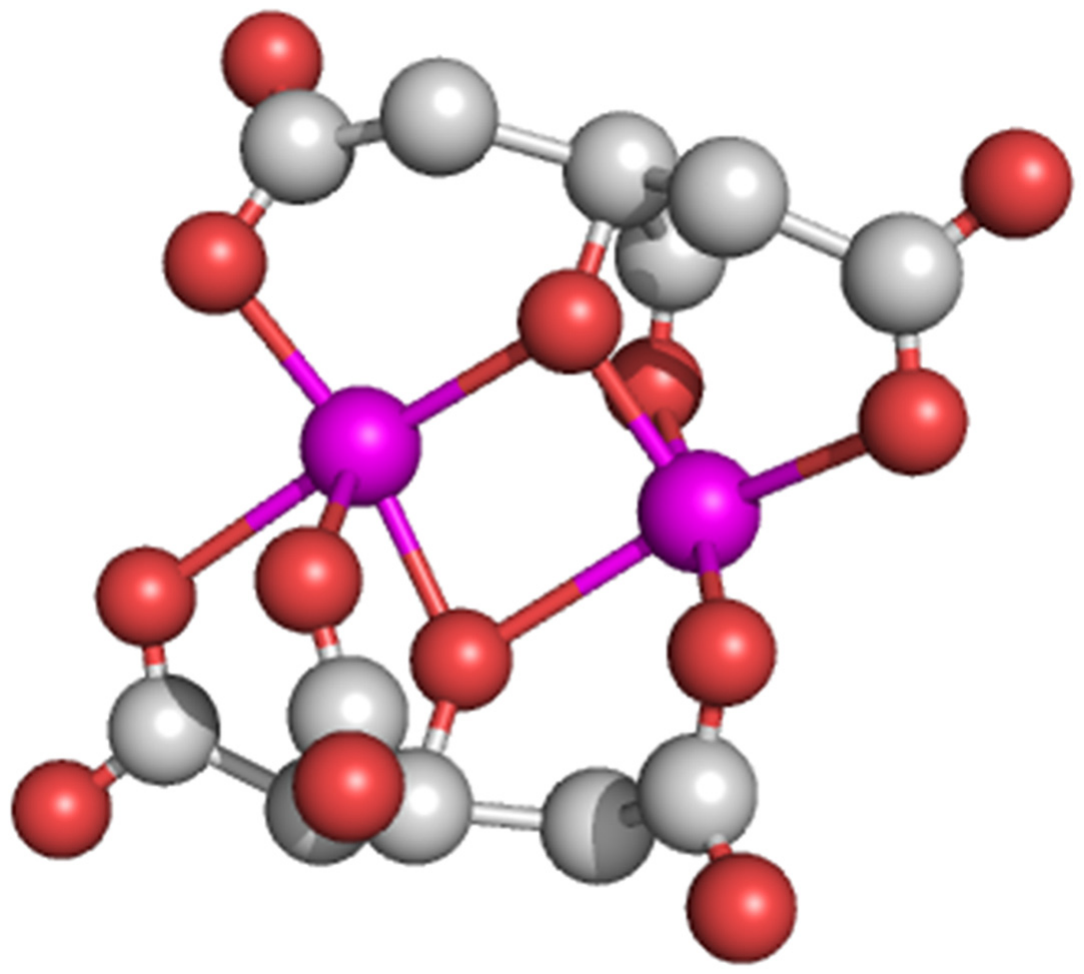
Structure of ferric citrate Structure of (Fe^3+^citrate)_2_ as determined in the FecA crystal structure (Ferguson *et al*. 2002, Yue *et al*. 2003). Color code: C in light grey, O in orange. and Fe in pink.

The iron transport system of *E. coli* is synthesized in response to FeCit in the growth medium (Frost Rosenberg 1973). Based on its complexation with iron (Fig. 2) citrate acts as a kind of siderophore. At high concentrations (1 mM) FeCit solubilizes Fe^3+^ and donates the metal to cells. Besides citrate, *E.coli* K-12 makes use of four other iron transport systems with the carriers aerobactin (Gross *et al*. 1985), ferrichrome (Fecker and Braun 1983), copogen (Hantke 1983), and enterobactin (Raymond *et al*. 2003), but these do not respond to extracellular signals and differ in their regulation. The presence of different iron transport systems with specific control mechanisms in a single strain of bacteria reflect the variety of iron sources in bacterial environments. The FeCit transport system has been found in ∼20% of the *E. coli* strains analyzed thus far and was probably acquired by horizontal gene transfer, as suggested by the accumulation of insertion sequence elements, inversions, and deletions in the region of *fec*, encoding the FeCit transport genes. These features have made it difficult to clone the *fec* genes (Pressler *et al*. 1988, Staudenmaier *et al*. 1989). In *E. coli* strains that elicit mastitis in Australian cows, the *fec* transport system acts as a virulence factor (Blum *et al*. 2018). The citrate concentration in milk (8 mM) is sufficient to form a FeCit transport system that can compete with lactoferrrin-bound iron.

FecA is the OM protein of the FeCit transport and signaling system and a small amount is constitutively present in the OM. In addition to transporting FeCit into the periplasm (Hancock *et al*. 1976, Hussein *et al*. 1981, Wagegg and Braun 1981), FecA signals the presence of FeCit in the growth medium (Zimmermann *et al*. 1984). In response to FeCit, the synthesis of FecA is up-regulated such that it becomes a major protein (∼80 000 molecules) of the OM and is thus able to trap the rare FeCit molecules in the growth medium. Of the various possible FeCit structures (Pierre and Gautier-Luneau 2000, Banerjee *et al*. 2016), iron dicitrate is the form that stimulates growth (Hussein *et al*. 1981). Analyses of the crystal structure of FecA with attached FeCit (Ferguson *et al*. 2002, Yue *et al*. 2003) revealed the structure of FeCit to be (Fe^3+^ citrate)_2_ (Fig. 2). FecA discriminates in its response between three loading states: unloaded, loaded with FeCit, and loaded with citrate Only the loading with FeCit strongly changes the structure of FecA (Fig. 1) (Ferguson *et al*. 2002, Yue *et al*. 2003) such that the large extracellular loops, in particular loops 7 and 8, close the external pocket. Binding of citrate without Fe to a similar site on FecA does not elicit loop movements.

The ligand-induced allosteric transitions resulting from the binding of FeCit to FecA is propagated through the OM by the plug domain of FecA. The structural changes extend from the extracellular space to the periplasm and involve the FecA signaling domain, located at the N terminus between the signal and plug domains. The signaling domain of FecA extends from residue 1 to residue 79 of the mature protein and differs from the signal domain, located between residues 1 and 33 of the precursor protein that mediates FecA secretion. While the signaling domain is not detected in the crystal structures of FecA, NMR studies of the isolated domain revealed a well-defined folded domain that is highly flexible relative to the β-barrel (Garcia-Herrero and Vogel 2005), which explains why it is not visible in FecA's crystal structure (Ferguson *et al*. 2002, Yue *et al*. 2003). The signaling domain construct used in the NMR study contained a flexible N-terminal tail (residues 75–96) that was completely unstructured in the NMR solution structure. In the crystal structures of FecA and FecA loaded with citrate, a small alpha-helix occurs at residues E94–V98 that is absent in the crystal structure of FecA loaded with FeCit. Upon binding of FeCit to FecA the helical region becomes disordered, which expands the range of motion of the signaling domain. This region harbors the TonB box, i.e., the site of FecA interaction with TonB. The FecA-TonB interaction presumably opens the pore to allow FeCit to enter the periplasm and affects the interaction between the signaling domain and the FecR regulatory protein (Fig. 1). Allosteric transitions in FecA involve structurally protein changes and no chemical reactions. Extracellular and periplasmic pockets of FecA undergo FeCit-mediated conformational changes. An evolution-based statistical analysis identifies a structurally connected network of residues linking distal functional sites in FecA (Ferguson *et al*. 2007). The free-energy change induced by FeCit binding at the cell surface is coupled with long-range conformational changes at the periplasmic surface. The plug domain closes the pore and establishes a functional connectivity within the network formed by the barrel and plug and the signaling domain (Ferguson *et al*. 2007).

Deletion of the signaling domain abolishes FecA regulation but FecA transport is retained (Kim *et al*. 1997). The addition of a surplus of separately synthesized signaling domain to the periplasm inhibits induction of *fecA* gene transcription but not transport, by competing with the binding of the signaling domain of intact FecA to FecR (see below). Since signaling requires the same components as transport -i.e., FeCit bound to FecA, structural changes in FecA and movement of the TonB box, as well as energization by the TonB ExbB ExbD complex—the separation of signaling from transport is difficult to achieve experimentally. Single-site mutations usually affect both functions (Härle *et al*.^1995^ Sauter *et al*. 2003). In transport-negative missense mutants constitutive *fecA* transcription is typically low. A particular interesting *fecA* mutant constitutively transcribes *fecA-lacZ* in a *tonB* mutant but requires TonB for FeCit transport (Härle *et al*. 1995). Since the activity of TonB is dependent upon an electrochemical potential, dissipation of the latter by CCCP treatment should not affect signaling by this mutant. Indeed, the level of *fecA-lacZ* transcription is not reduced by CCCP (Kim *et al*. 1997). Thus, according to the findings obtained with this mutant, the interaction of TonB with FecA for signaling is not identical to the interaction of TonB with FecA for transport.

Transport-negative but signaling active *fecA* mutants indicate transcriptional control by FeCit attached to FecA at the cell surface. Additional experimental findings support this conclusion: (i) FeCit is small enough (MW 488) to diffuse through porins at a rate sufficient to support growth. Indeed, *fecA* deletion mutants grow on 50 mM citrate and, if the *fecBCDE* genes required for transport across the cytoplasmic membrane are overexpressed, exhibit low-level iron transport capacity (30% of that of the wild-type). Under these conditions, *fec* gene transcription is not initiated (Härle *et al*. 1995). (ii) The same concentration of FeCit (0.1 mM) that induces the transport system is also required to maintain it. Enhanced transport does not lower the critical concentration of FeCit. (iii) While an intracellular citrate concentration in the range of 2–12 mM would be expected to induce *fec* transcription, provided there is enough free Fe^3+^ to form FeCit, this is not the case. d) Fe^3+^fluorocitrate and Fe^3+^phosphocitrate are poor transporters of iron but both induce *fec* transcription (Hussein *et al*. 1981). (iv) FeCit initiates *fec* gene transcription in *fecCDEF* mutants unable to import FeCit into the cytoplasm. These findings indicate the initiation of *fec* transcription through FecA in the absence of FeCit uptake.

### Signaling by the PupA and PupB outer membrane transport proteins of *Pseudomonas putida*

Siderophores are synthesized under iron-restrictive concentrations, characteristic of the habitats of *Pseudomonas*, in which under oxic conditions and a pH of ∼7 Fe^3+^ forms insoluble Fe^3+^-hydroxy precipitates. In addition, Pseudomonas hosts actively restrict available iron as a defense strategy. To counteract this environmental iron deficiency, Pseudomonas synthesizes siderophores that are secreted and form Fe^3+^carriers. Moreover, *Pseudomonas* strains use not only their own but also the siderophores of other strains (Table 1) (Beare *et al*. 2003, Llamas and Bitter 2006, (Llamas *et al*. 2014), Otero-Asman *et al*. 2019, Visca *et al*. 2002). Early insight into the transcriptional regulation of ferric siderophore transport genes was obtained in an elegant study of two siderophores of *Pseudomonas putida* (now *P. capeferrum*), pseudobactin BN8, and pseudobactin 358 (Koster *et al*. 1994). Promoter activities were determined by fusion of the studied promoters to the *E. coli* β-galactosidase *lacZ* gene as the reporter. Synthesis of the OM protein PupB is induced by BN8 which is transported by PupB across the OM. BN8-dependent PupB synthesis is regulated by two proteins, PupI and PupR, which display 42.8% and 36.6% identity with FecI and FecR, respectively. Pseudobactin-dependent transcription activation does not require BN8 transport by PupB into the periplasm since a *pupB* mutant with 50% of wild-type BN8 uptake does not initiate *pupB-lacZ* expression. Binding of BN8 to PupB alone also fails to elicit transport gene transcription. Instead, both PupB-mediated signaling and BN8 transport rely on the energy provided by the TonB, ExbB, ExbD system. With the exception of transcription initiation by PupI without PupR, the regulation of signaling through BN8 is similar to that through FeCit, although the latter requires FecR to activate FecI. However, transcription activity in response to PupI alone is less than that in response to PupI and PupR, which suggests both inhibitory and activating functions for PupR (Koster *et al*. 1994). In fact, the *pupR* deletion gene used in these experiments was constructed by replacing an internal fragment of the *pupR* gene by an omega cassette. The resulting construct may have retained a functional N-terminal fragment with pro-sigma activity, which explains the increased PupI activity despite the deletion of *pupR*.

**Table 1.** Cell surface signaling systems.^1^

Organism	Iron carrier	Outer membrane receptor	Sigma factor	Anti/Pro sigma factor
*E. coli*	Citrate	FecA	FecI	FecR
*P. aeruginosa*	Pyoverdine	FpvA	FpvI^2^, PvdS^2^	FpvR
*P. aeruginosa*	Ferrioxamine B	FoxA	FoxI	FoxR
*P.aeruginosa*	Ferrichrome	FiuA	FiuI	FiuR
*P. putida* ^5^	Aerobactin	IutA	IutY^4^	IutY^4^
*P. putida*	Pseudobactin BN8	PupB	PupI	PupR
*P. putita*	Pseudobactin 358	PupA	PupI	PupR
*S. marcescens*	heme	HasR	HasI	HasS

1 The table contains a selection of well-studied systems that are discussed in this review. Many more exist, for example 14 in *P. aeruginosa* (Koebnik 2005).

2,3 Two ECF sigma factors regulate pyoverdine synthesis and transport. ^4^The YutY polypeptide is composed of an N-terminal sigma factor and a C-terminal anti-sigma factor. ^5^This strain was renamed *Pseudomonas capeferrum*

**Figure 3.**
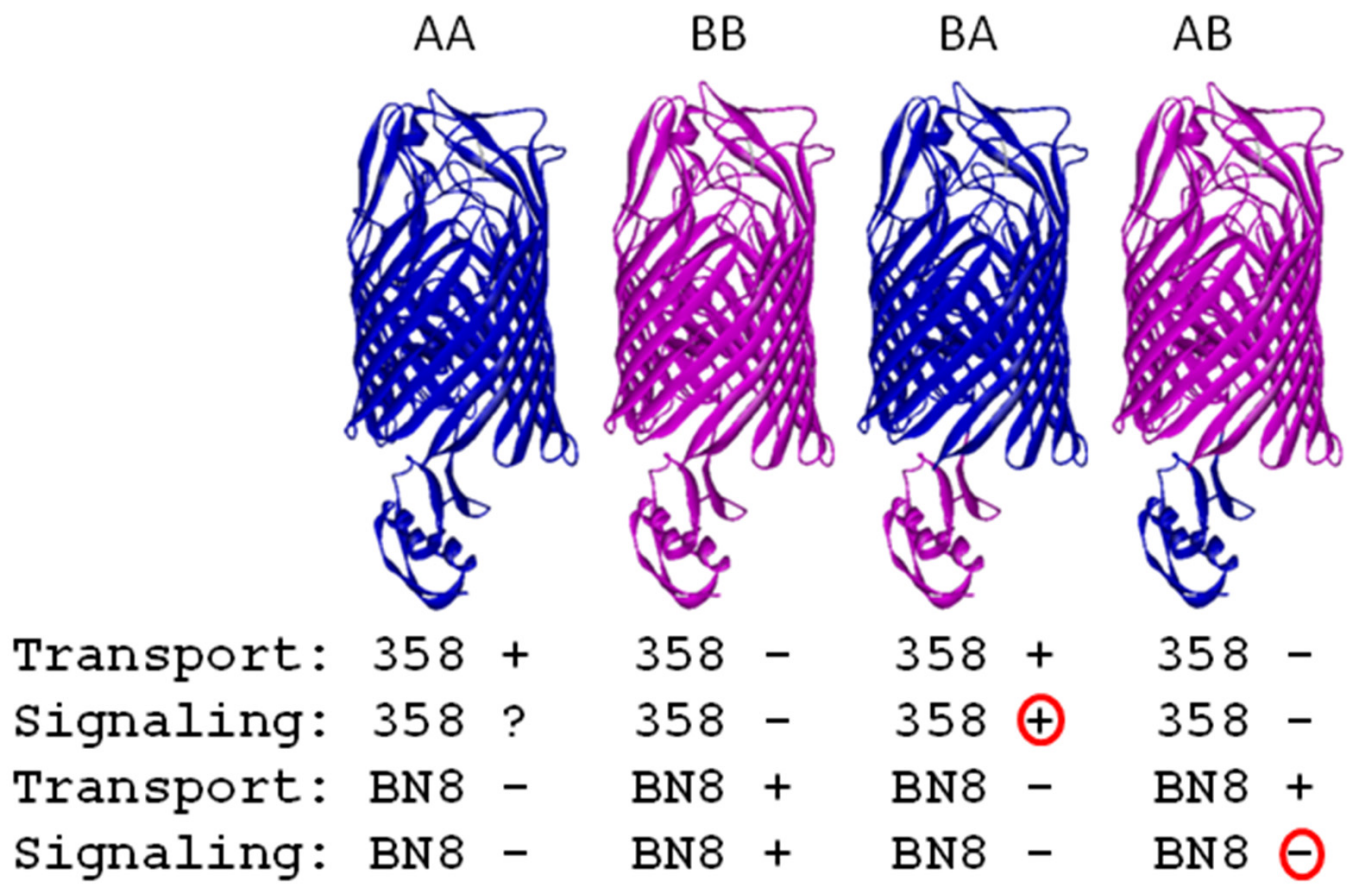
Different substrate specificities for pseudobactin signaling and transport Illustration of the chimeric PubA and PupB β-barrels to which heterologous PupB and PupA signaling domains were fused, resulting in PupBA and PupAB proteins. Transport and signaling of the chimeric proteins in response to pseudobactins BN8 and 358 is indicated; PupA is specific for pseudobactin 358 and PupB for pseudobactin BN8. The signaling and transport specificity of the BA protein is determined by the A β barrel. The transport specificity of the AB protein is determined by the B β barrel but the chimera no longer signals (Koster *et al*. 1994). ? indicates not known.

Before the crystal structure of an OM siderophore receptor was determined (Ferguson *et al*. 1998, Locher *et al*. 1998), a topology model for the folding of PupB in the OM was proposed (Koster *et al*. 1994). It predicted an N-terminal domain that is exposed to the periplasm and larger than the N-terminal domains of OM proteins without signaling activity. To assign a function to this N-terminal extension, a chimeric receptor was constructed in which the first 86 amino acids of mature PupB were replaced by the corresponding sequence of the PupA OM receptor (Fig. 3). PupA transports pseudobactin 358. The PupAB chimera transported BN8, but BN8 no longer induced *pupB-lacZ* transcription (Koster *et al*. 1994). However, a PupBA chimera was able to transport pseudobactin 358 and to induce pseudobactin-358-dependent *pupB-lacZ* transcription. In PupBA, the original transport and induction specificity of PupA was retained but *pupB-lacZ* transcription was elicited through the PupB signaling domain. These results demonstrated the independence of transport and signaling specificities, as also shown in FecA mutants capable of transport but not signaling. Koster *et al*. concluded that “the stimulus to which the two component system responds is not the ferric siderophore complex itself, but a signal that is transduced by the receptor upon transport of its substrate”. This key insight was verified by subsequent studies of the OM-mediated transcriptional control of Fe^3+^siderophore transport and siderophore synthesis genes.

### Fe^3+^pyoverdine transport and signaling by the FpvA protein of *Pseudomonas aeruginosa*

The best studied OM protein of *Pseudomonas aeruginosa* is FpvA, which transports ferric pyoverdine and regulates pyoverdine synthesis and transport (Folschweiller *et al*. 2000). The crystal structure of FpvA loaded with ferric pyoverdine at 2.7Å resolution (Wirth *et al*. 2007) reveals not only the β-barrel with the plug domain in its lumen but also the signaling domain. By contrast, the signaling domain is not seen in the crystal structure of FpvA loaded with iron-free pyoverdine (Cobessi *et al*. 2005, Jensen *et al*. 2015). The open conformation of the latter structure is similar to FecA loaded with iron-free dicitrate (Ferguson *et al*. 2002, Yue *et al*. 2003). Despite the low sequence identity (19.7%) between FpvA and FecA, their crystal structures include a common fold in the β-barrel and plug domains. Moreover, the basic structures are conserved not only among the two proteins but also in all TonB-dependent OM transporters. In all OM proteins loaded with Fe^3+^siderophores, the loop that contains the TonB box and connects the signaling domain to the plug is not defined in the electron density map. The high flexibility of this loop is probably necessary for both signal transduction and transport across the OM. Fe^3+^pyoverdine binding to FpvA induces the transcription of pyoverdine synthesis and Fe^3+^pyoverdine transport genes (Schalk *et al*. 2009). In the absence of Fe^3+^pyoverdine, the three-stranded β-sheet of the signaling domain binds to the TonB box of FpvA; for Fe^3+^pyoverdine transport, TonB binds to the TonB box and displaces the signaling domain. Specifically, the structural transitions in FpvA that are induced upon Fe^3+^pyoverdine binding lessen the affinity of the signaling domain for the TonB box such that TonB binding is favored. Following its displacement from the TonB box, the signaling domain is free to interact with the FpvR regulatory protein, which in turn is fragmented to release the sigma factors FpvI/PvdS (discussed in chapter 5) that initiate the transcription of pyoverdine synthesis and Fe^3+^pyoverdine transport genes. As in *E.coli*, the reactions of *Pseudomonas* OM proteins that mediate transport and signaling are probably similar, including a conformational change in those proteins upon binding of the Fe^3+^ carriers to the surface-exposed residues. However, while for transport the plug must move to accommodate diffusion of the large Fe^3+^siderophores, a similar structural transition is likely unnecessary for signaling.

## Signal transfer across the periplasm

### Interaction of the signaling domain with the sigma regulatory protein

**Figure 4.**
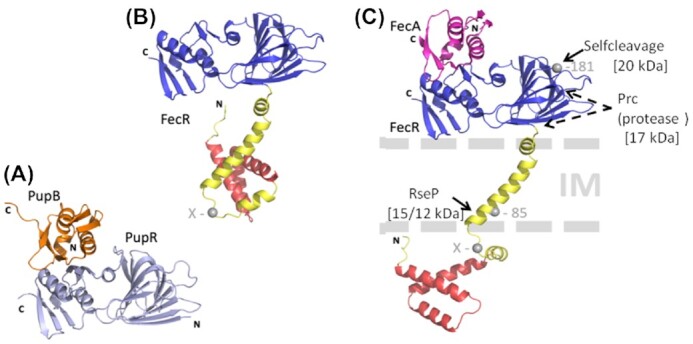
Structures of signaling domains (**A**) Crystal structure of the PupB signaling domain attached to the PupR periplasmic domain (Jensen *et al*. 2020). (**B**) Structure of the FecR periplasmic domain as predicted by the AlphaFold Protein Structure Database (Jumper *et al*. 2021). (**C**) Structure of the FecA signaling domain attached to the modified structure of the FecR periplasmic domain. The periplasmic domains are colored blue, the cytosolic 3-helical domains red. Due to the absence of the membrane in the prediction process, the cytosolic domain was predicted in a position that is expected to be occupied by the membrane, although no specific contacts are predicted between the 3-helical domain and the transmembrane helix (yellow). To resolve the conflict with the inner membrane shown in B), the orientation of the cytosolic domain was altered by a rotation about residue 76 (indicated by an “X” in panels B and C), which resides in a flexible loop before the predicted transmembrane region. Further, based on a superposition to the PupR:PubB complex (A), the FecA N-terminal signaling domain (PDB: 2D1U) was modeled onto the FecR periplasmic domain as shown in C). The proteolytic cleavage site at residue 181 is indicated. Residue 85 was deduced from localization studies with FecR-BlaM fusion proteins but prediction programs favor residue 82.The arrows indicate unknown cleavage sites of the FecR domain by the protease Prc and possibly by additional proteases in the periplasm and by the RseP protease in the cytoplasmic membrane (IM). FecR fragments of 20-kDa, 15-kDa, and 12-kDa are formed. N, N-terminal, C, C-terminal ends of the polypeptide chains.

The signal elicited by FeCit through binding to FecA is transmitted to the sigma regulatory protein FecR in the periplasm. Experimental localization studies of FecR, using fusions of the ß-lactamase BlaM with various positions of FecR identified its N-proximal portion in the cytoplasm, a transmembrane region (residues 85–100) in the cytoplasmic membrane, and the C-proximal region in the periplasm (Kim *et al*. 1997, Welz and Braun 1998) (Fig. 4C). The FecA signaling domain (residues 1–79) interacts with the C-proximal end of FecR (residues 101–317), as demonstrated by the use of a bacterial two-hybrid system (Enz *et al*. 2003a), the adherence of FecA on a column loaded with FecR (Enz *et al*. 2000), the characterization of inactive *fecA* and *fecR* mutations, and the suppression of *fecR* mutations by *fecA* mutations (Stiefel *et al*. 2001, Enz *et al*. 2003a).

A high-resolution structure of the PupB signaling domain (residues 45–130) co-crystallized with the complete C-terminal periplasmic fragment (residues 110–324) of PupR (Fig. 4A) was recently reported and provided detailed structural insights into the interaction of the two proteins (Jensen *et al*. 2020). The formation of a 1:1 complex between the 45–130 portion of PupB and the 110–324 fragments of PupR is not associated with strong changes in the secondary structure of the PupR signaling domain. The C-terminal PupR fragment consists of two subdomains, comprising residues 110–238 and 250–324, connected by a flexible linker. Even in the absence of signaling the two subdomains spontaneously bind to the PupB signaling domain and form a stable structure. Without the signaling domain the periplasmic domain is flexible and rapidly degraded due to its highly dynamic and therefore protease-sensitive structure. Binding to the PupB signaling domain requires both subdomains, which in turn stabilizes the subdomains. These findings are important for the understanding of the signal transfer across the periplasm into the cytoplasm. Obviously, signaling dissociates the regulatory proteins from the signaling domains and renders the regulatory proteins sensitive to proteases.

## Interaction of the N-proximal region of the sigma regulatory protein with the sigma factor initiates transcription

In the *E.coli fec* system, under physiological conditions (i.e., chromosomally encoded genes) the activity of the FecI ECF sigma factor depends entirely on the presence of the pro-sigma FecR regulatory protein. While FecI is inactive in the absence of FecR, in response to FecR it directs the RNA polymerase to the promoter in front of the *fecA* gene (Angerer *et al*. 1995, Enz *et al*. 1995, Angerer and Braun 1998), as demonstrated in *E. coli* K-12, *E. coli* B, *K. pneumoniae*, and *Photorhabdus luminescens* (Mahren *et al*. 2005, Ochs *et al*. 1995). Only highly overexpressed FecI exhibits residual activity in the absence of FecR (Ochs *et al*. 1995). This dependence between FecI and FecR is unique among sigma regulatory proteins with anti-sigma activity in which active sigma factors are released following hydrolysis of the anti-sigma factors. Control of a single promoter, in front of *fecA* and its low abundance, less than one molecule per cell in the unrepressed state (Maeda *et al*. 2000) are additional special features of FecI.

**Figure 5.**
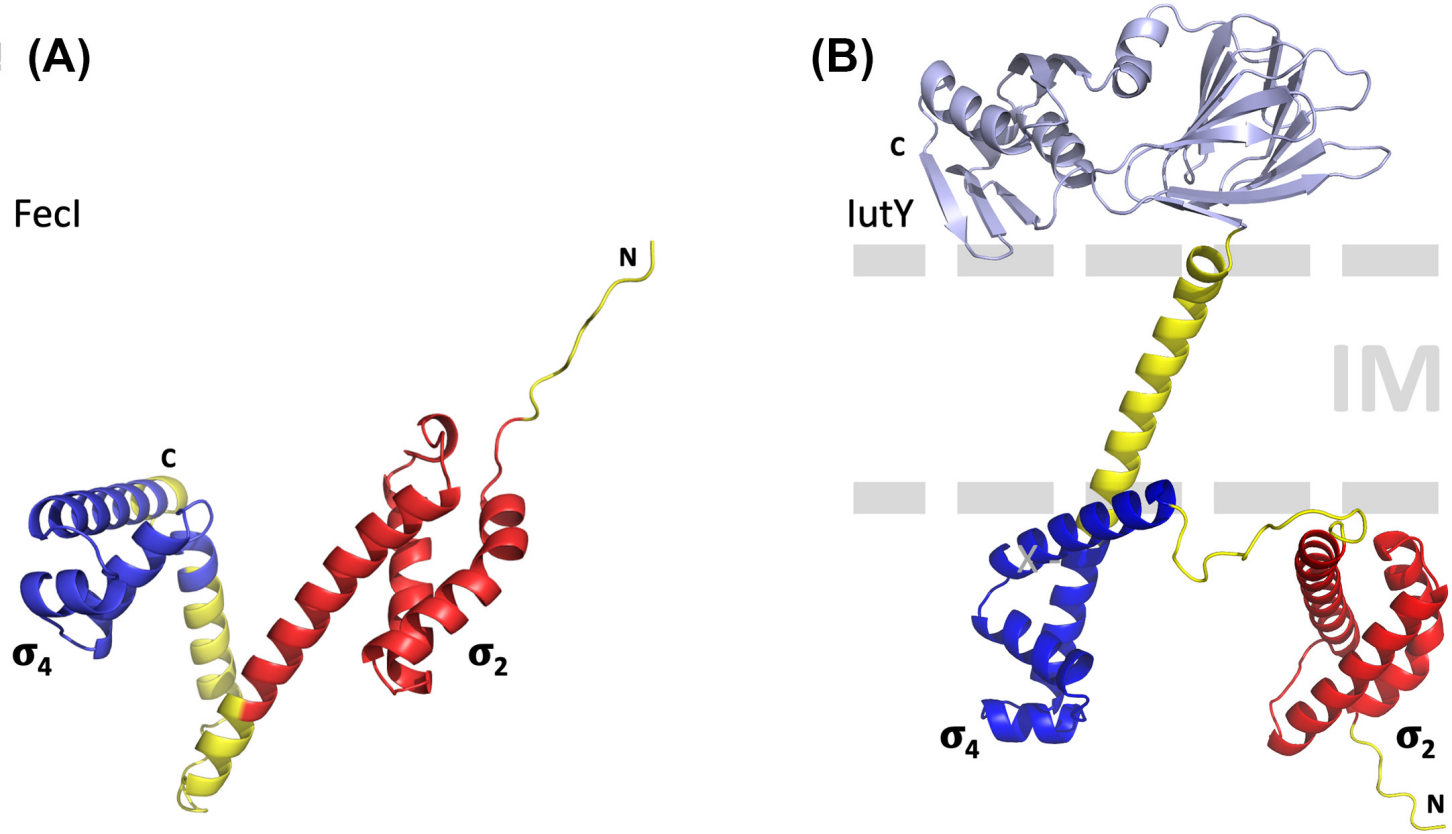
FecI and IutY structure predictions. (**A**) FecI structure prediction from the AlphaFold Protein Structure Database. The σ_2_ and σ_4_ domains are marked according to the sequence alignment from Lonetto *et al*. (1994). Of note, there are no interactions formed between the helices linking the σ_2_ and σ_4_ domains, implying that their connection is flexible. (**B**) IutY structure prediction. Depicted is the 1st-ranked model from an AlphaFold structure prediction (Jumper *et al*. 2021), together with a schematic of the inner membrane (IM). The cytoplasmic part of IutY is predicted to fold into two domains that closely resemble the σ_2_ and σ_4_ domains of FecI, respectively colored red (IutY residues 8-84) and blue (100-164). The periplasmic portion of IutY (light blue) closely resembles the periplasmic domain of FecR.

The structure of FecI was estimated with the AlphaFold prediction program (Fig. 5A) It consists of two independently folded globular domains connected by a flexible linker. It resembles the structure of the σ^E^ stress sigma factor with the σ_2_ and σ_4_ domains which contains in the determined structure the co-crystallized fragment of the RseA anti- σ factor sandwiched between σ_2_ and σ_4_ (Campbell *et al*. 2003). This FecI structure is typical for sigma factors of the σ^70^ family.

An *in vivo* deletion analysis using a bacterial two-hybrid system showed that the N-terminal cytoplasmic region of FecR interacts with σ_4_ of FecI (Fig. 5) (Mahren *et al*. 2002). In randomly generated missense mutants carrying mutations localized in σ_4_, the interaction of FecR with FecI is impaired. The physical binding of the N-terminus of FecR to FecI was demonstrated using FecR tagged at its C-terminal end with (His)_6_ or at its N-terminal end with (His)_10_. Only the former was able to bind FecI because (His)_10_ interfered with FecR binding (Enz *et al*. 2000). The FecR/FecI interaction is specific, since FecR_1-85_ does not bind to PA2468, the FecI equivalent in *P. aeruginosa* (Mahren *et al*. 2002).

### The N-terminal ends of regulatory proteins bind to the sigma factors

**Figure 6.**
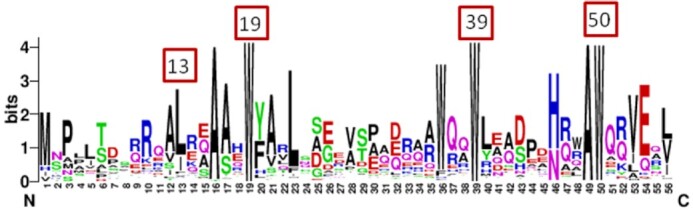
Sequence Logo of FecR homologs In a PSI-Blast about 100 homologs to *E. coli* FecR were found (Gabler *et al*. 2020) and a comparison in the form of a sequence logo is shown (Crooks *et al*. 2004). The highly conserved tryptophan and leucine residues are indicated.

A precise localization of the site by which FecR binds to FecI was achieved already in 1995, in a study using two chromosomal *fecR* nonsense mutants involving codons 82 and 202 (full size: 317 residues) (Ochs *et al*. 1995). Cells carrying either mutation were able to synthesize β-galactosidase encoded by *fecB-lacZ* independent of FeCit. The N-terminal FecR fragments confer constitutive FecI activity. A systematic study with plasmid-encoded 3’-truncated *fecR* genes revealed the FeCit-independent induction of *fecA-lacZ* transcription by FecR59 (residues 1–59), FecR61, FecR65, FecR68, and FecR116 but not by FecR46 and FecR56 (Ochs *et al*. 1995). Thus, the inducing fragment of FecR comprises residues 1–59. Randomly generated induction-negative FecR mutants contained amino acid replacements at L13Q, W19R, W39R, and W50R (Stiefel *et al*. 2001). All of these amino acids are located in the FecI-inducing 1–59 region and are highly conserved in all FecR-like sequences (Fig. 6) (Stiefel *et al*. 2001, Braun and Mahren 2005). In PupR of *Pseudomonas*, two of the tryptophan residues, W20 and W40 (W19 and W39 in FecR), are located in the inner core of the protein's cytoplasmic fragment and contribute to its stability (Jensen *et al*. 2015). Specific binding of the FecR N-terminal fragments to FecI was demonstrated with the LexA/sulA two hybrid system (Dmitrova *et al*. 1998). The combination of FecI_1-173_ with the FecR deletion derivatives FecR_1-58,_ FecR_1-85_, and FecR_9-85_ repressed the transcription of *lacZ* under the control of the altered *sulA* operator, whereas FecR_1-38_ and FecR_19-85_ failed to repress *lacZ*, presumably because they were unable to bind to FecI. The N-terminal transcription-active FecR fragments associated with FecI, whereas the transcription-inactive FecR fragments were unable to bind FecI. These data indicated that the FecR region from residues 9 to 59 interacts with FecI and thereby converts it into an active sigma factor.

### *Pseudomonas* sigma regulatory proteins and their fragmentation

The regulatory site of *Pseudomonas* anti-sigma factors has also been localized to the N-proximal region. FpvA loaded with pyoverdine suppresses the anti-sigma activity of the FpvR regulatory protein, resulting in active FpvI and PvdS sigma factors (Table 1) (Beare *et al*. 2003, Spencer *et al*. 2008). A genetically constructed 67-residue N-terminal fragment of FpvR binds to and inactivates the C-terminal regions of FpvI and PvdS (Redly and Poole 2005). A reduced binding of mutant FpvR correlates with a substantial drop in the amounts of FpvI and to a lesser extent of PvdS, suggesting that FpvI and PvdS are stable when bound to FpvR but in their free, active form are prone to turnover. This rapid turnover of sigma factors ensures that the continued activation of the target gene is dependent upon their ongoing synthesis and allows the system to quickly respond to the absence of inducer. Analyses of FpvI mutants showed that the FpvI structures required for anti-sigma factor binding and those required for promoter binding are the same (Redly and Poole 2005). In contrast to FecI activation by the N-terminal fragment of FecR the N-terminal fragment of FpvR inhibits FpvI and PvdS.

### Pro-sigma factor regulation of the sigma factors FecI, FoxI, and FiuI

In *E. coli fecABCD* gene transcription requires the presence of the FecR regulatory protein to which we refer to as pro-sigma factor. The N-terminal sequence from residue 9-59 stimulates the FecI sigma factor. In *Pseudomonas*, ferrioxamine B and ferrichrome uptake are mediated by the Fox and Fiu systems, which include the OM proteins FoxA and FiuA, respectively (Table 1). The regulatory proteins FoxR and FiuR act as anti-sigma factors in that they inhibit the sigma factors FoxI and FiuI (Bastiaansen *et. al*.2015a). However, the cytoplasmic portions of FoxR_1-93_ and FiuR_1-91_ exhibit pro-sigma activity (Mettrick and Lamont 2009). In the absence of chromosomally expressed FoxR protein, overproduced plasmid-encoded FoxR_1-93_ induces *foxA* gene expression irrespective of the presence of desferrioxamine B. The same result is obtained with FiuR, the anti-sigma factor of FiuI. FiuR_1-91_ enhances *fiuA* expression by a *fiuR* mutant in the absence of ferrrichrome. However, in the absence of the cognate siderophores, the overexpression of complete *foxR* and *fiuR* in wild-type bacteria reduces gene expression, consistent with the anti-sigma activities of these proteins.

## Fragmentation of the sigma regulatory protein

The shortest genetically constructed N-terminal fragment of FecR that stimulates *fecA* expression is 51 residues long (Ochs *et al*. 1995). This fragment and all other transcription-stimulating N-terminal FecR fragments do not require FeCit for induction. SDS gel electrophoresis of wild-type FecR revealed a dominant N-terminal 20-kDa fragment derived from the cleavage of FecR between glycine (Gly) 181 and threonine (Thr) 182 (Wriedt *et al*. 1995). The 20-kDa fragment was accompanied by a 15-kDa fragment (Wriedt *et al*. 1995, Welz and Braun 1998) (Fig. 4C). Pulse-chase experiments with radiolabeled methionine demonstrated further degradation of the 15-kDa fragment to a 12-kDa fragment (Welz 1998). However, the 20-kDa fragment is not essential for FecR activation (Wriedt *et al*. 1995). Neither the 20-kDa nor the 15-kDa fragment was produced by two *fecR* mutants in which *fecA-lacZ* transcription was induced to 80% and 50% of the wild-type level. None of the multiple amino acid replacements in these FecR derivatives were at the Gly-Thr cleavage site. The resistance of the Gly-Thr bond indicates structural alterations of FecR that prevent its cleavage and reduce its activity. The 20-kDa fragment was also prominent in FecR purified from cells grown without FeCit induction (Enz *et al*. 2000). Its formation was independent of signaling and presumably results from chemical self-cleavage of the Gly-Thr bond. Internal deletion derivatives of FecR devoid of the Gly181-Thr182 bond, such as FecR∆144-223 and FecR∆153-187, strongly activated *fecA-lacZ* transcription without the involvement of FeCit. The mechanism leading to autoproteolytic Gly-Thr cleavage was elucidated in studies of the response of *Pseudomonas aeruginosa* to ferrioxamine B (Bastiaansen *et al*. 2015a). Prior to ferrioxamine B stimulation, FoxR fragments of 21 and 15 kDa are generated by non-enzymatic cleavage of the protein between Gly191 and Thr192, through an N-O acyl rearrangement in which an ester bond that forms between the two amino acids is subsequently hydrolyzed.

Further insights into FecR fragmentation were obtained in studies of the LacZ activities resulting from the induction of *fecA-lacZ* transcription by N-terminal FecR fragments fused to the *blaM* gene. FecR as short as 61 residues (FecR61-BlaM) was shown to stimulate *fecA-lacZ* transcription. FecR61 is about the same size as the FecR fragment comprising residues 9–59, which stimulates *fecB-lacZ* transcription in the absence of FeCit. The highest level of transcription activation among the fusions generated from FecR residues 61–301 was achieved with the FecR81-BlaM fusion which presumably resembles the natural substrate from which the FecI-activating FecR fragment is released. SDS-PAGE showed that 20-kDa and 15-kDa fragments were derived from BlaM fusions in which FecR was 230 residues and longer. Shorter, more active FecR-BlaM fusions lacked the Gly181-Thr182 bond. BlaM fused to the FecR fragments does not abolish FecR fragmentation. Fragmentation tolerates heterologous substitution of large portions of the C-terminal part of FecR without inhibition of the formation of the inducing N-terminal fragment and of the 20 kDa fragment. These results demonstrate the dispensability of the 20-kDa and 15-kDa fragments and suggest different proteolysis pathways that finally yield the N-terminal FecR transcription-activating fragment.

Several *Pseudomonas* systems were studied to identify the proteolytic pathways resulting in fragments that inactivate anti-sigma factors (Table 1). Fe^3+^pyoverdine attached to the FpvA receptor triggers FpvR hydrolysis by RseP. In the presence of pyoverdine, the anti-sigma factor FpvR is completely hydrolyzed, resulting in an active FpvI sigma factor that initiates the transcription of pyoverdine synthesis and transport genes (Draper *et al*. 2011). An *rseP* mutant is incapable of *fpvA* gene transcription (Bastiaansen *et al*. 2014). In a pyoverdine-deficient mutant, a 20-kDa N-terminal fragment of FpvR (full size: 37 kDa) is formed that disappears within 30 min after the addition of pyoverdine to the mutant culture (Draper *et al*. 2011). The pyoverdine-positive parent does not contain the 20-kDa fragment. FpvA loaded with Fe^3+^pyoverdine signals the hydrolysis of FpvR. In the presence of FpvR and the 20-kDa fragment, transport genes are not transcribed.

The binding of Fe^3+^ferrioxamine B to the FoxA OM protein triggers the transcription of ferrioxamine transport genes (Table 1). In ferrioxamine B-treated cells, two FoxR fragments are formed: an N-terminal 21-kDa fragment and a C-terminal 15-kDa fragment. The former is further degraded by the RseP protease to a 12-kDa N-terminal tail, which may be the final degradation product released from the cytoplasmic membrane into the cytoplasm, where it relieves the anti-sigma activity of FoxR. However, the 21-kDa and 15-kDa fragments can also arise prior to ferrioxamine B-stimulation by non-enzymatic cleavage between Gly191 and Thr192 (Bastiaansen *et al*. 2015a). Replacement of Gly191 and Thr192 by other amino acids prevents self-cleavage but retains approximately 70% of the ferrioxamine B-dependent activation of *foxA-lacZ* transcription. Self-cleavage of FoxR is not essential for FoxI activation. The C-terminal part of FoxR is required for the response to ferrioxamine B. The C-domain inhibits the N domain (Bastiaansen *et al*. 2015b). Overexpression of the cytosolic N-domain results in a 200-fold increase in *foxA* transcription. The N-domain fragment in the cytoplasm and the C-domain fragment in the periplasm act in concert to activate FoxI-mediated *foxA-lacZ* transcription.

Studies of the transcriptional regulation of aerobactin transport genes revealed the proteases involved in the fragmentation of an anti-sigma factor (Bastiaansen *et al*. 2014, Bastiaansen *et al*. 2017). Transcription activation requires the periplasmic Prc protease and the membrane-inserted RseP protease. Aerobactin is a siderophore that supplies iron to *P. putida* (Table 1). The binding of iron-loaded aerobactin to the OM IutA receptor triggers transcription of the *iutA* gene. Transport gene transcription is regulated by a unique protein that consists of a fusion between a cytosolic ECF sigma factor (σ^IutY^) and a periplasmic anti-sigma factor, with the two components separated by a single transmembrane domain (Fig. 5B). In response to aerobactin binding to IutA, the IutY protein is cleaved sequentially: first, C-terminal-processing Prc degrades the periplasmic anti-sigma domain, followed by RseP, which removes the transmembrane domain and thereby generates transcriptionally active σ^IutY^. High levels of a 23-kDa N-terminal fragment of IutY (full size: 41 kDa) are present in aerobactin-treated cells and in cells overexpressing Prc. In *prc* mutants the fragment is absent and no aerobactin-induced *iutA* is observed (Bastiaansen *et al*. 2014, Bastiaansen *et al*. 2017). C-terminally truncated IutY variants constitutively transcribe *iutA-lacZ*. Shorter, but not longer derivatives (up to residue 236) do not require Prc. This agrees with the previously discussed results obtained with FecR, in which FecR-BlaM fusions ≥ 230 residues are cleaved to 20-kDa and 15-kDa fragments whereas no such fragments are generated by shorter variants. Regardless of the formation of the 20-kDa and 15-kDa fragments, FecR activates FecI. Apparently, formation of the 20-kDa fragment and its further cleavage to a 15-kDa fragment can be bypassed. IutY is processed by Prc between residues 259 and 374, which generates the substrate for RseP. This model probably also applies to FecR as well as to the ferrichrome FiuR and ferrioxamine B FoxR sigma regulatory proteins (Bastiaansen *et al*. 2017). However, in contrast to the aerobactin system, deletion of the *prc* gene reduces, but does not abolish ferrichrome and ferrioxamine B-mediated signaling. Under these conditions, Prc is probably replaced by other proteases.

### The proteases Prc and RseP cleave FecR

Experimentally created transcription-activating FecR fragments suggest in vivo fragmentation of FecR in response to the FeCit signal attached to FecA. Activating FecR fragments are probably excised by periplasmic proteases and then further processed in the cytoplasm (Fig. 4C), similar to the proteolysis of the RseA anti-sigma factor of *E. coli*, which regulates the activity of the stress response sigma factor σ^E^ (Brooks and Buchanan 2008). The periplasmic domain of RseA is cleaved first by the DegS protease and then in the cytoplasmic membrane by the RseP protease. However, mutants in *degS* and *rseA* do not reduce the level of FecA synthesis, as revealed by immunoblotting using anti-FecA antibodies. By contrast, mutants in *rseP* strongly reduce the amounts of FecA (Braun *et al*. 2005). RseP does not influence the constitutive synthesis of FecA elicited by the N-terminal FecR_1-85_ fragment, the size of which is close to that of the RseP degradation product (see below). Complementation of the *rseP* mutants by plasmid-encoded wild-type *rseP* restores FecA expression. Based on these observations, it was concluded that FecR is cleaved within or at the cytoplasmic membrane by RseP and that the cleavage product released into the cytoplasm activates FecI. The size of FecR_1-85_ may closely resemble the natural RseP-generated FecR activation fragment. It was proposed that ferric citrate induction changes the conformation of FecR such that it becomes sensitive to specific RseP cleavage (Braun *et al*. 2005). The cleavage product released into the cytoplasm activates FecI. As RseP is located in the cytoplasmic membrane (Akiyama *et al*. 2004), it is perfectly situated to create the FecR fragment that activates FecI.

In a recent study aimed at identifying additional cellular functions of the RseP protease, mass-spectrometry revealed lower levels of the FecA, FecB and FecD proteins in an inactive *rseP* point mutant relative to the levels in the *rseP* wild type (Yokoyama *et al*. 2021). In the *rseP* mutant, *fecA-lacZ* transcription was not elevated in response to FeCit, in contrast to the ten-fold higher induction of LacZ activity in the *rseP* wild-type. A comparison of the FecR protein pattern following SDS-PAGE revealed an N-terminally labeled 25-kDa band and a 15-kDa band in RseP wild-type cells whereas the RseP mutant cells contained a 17-kDa band. These bands were probably the same as those obtained from FecR preparations as described above, with their somewhat larger size resulting from their having been labeled at the N-terminus with a 3xFLAG tag. Interestingly, the 15-kDa and 17-kDa bands were observed only in cells grown in FeCit-containing medium. In pulse-chase experiments of RseP mutant cells with radioactive methionine, the FecR band disappeared within a 3-min chase whereas the amount of the 25-kDa fragment decreased and that of the 17-kDa product increased within 80 min. The rate of decrease of the 25-kDa fragment corresponded to the rate of increase of the 17-kDa fragment, indicative of a precursor-product relationship. In RseP wild-type cells, the 17-kDa fragment is progressively replaced by a 15-kDa fragment, which suggests RseP-mediated hydrolysis. Support for this possibility was obtained by estimating the sizes of the FecR degradation fragments based on a comparison of their electrophoretic mobilities with those of genetically constructed FecR C-terminal degradation products. The smallest fragment, FecR85 (85 residues), assumed a position similar to that of the 15-kDa fragment. Since residue 85 is close to the inner side of the cytoplasmic membrane, where RseP resides (Akiyama *et al*. 2004), the 15-kDa fragment arises by proteolytic cleavage of the 17-kDa fragment by RseP.

These findings identify a proteolytic pathway of FecR that generates the active FecR fragment but they do not exclude further fragmentation by cytoplasmic proteases to yield the final FecI-activating FecR product nor do they disclose the action of FeCit. In transport assays, the radioactive citrate label of FeCit did not accumulate in cells, unlike Fe (Hussein *et al*. 1981), which rules out FeCit transport into the cytoplasm. However, FeCit might be taken up into the periplasm, where Fe^3+^, or more likely Fe^2+^, would dissociate from citrate, which would then escape through porins during the washing procedure of the transport assay. According to this scenario, some FeCit would persist in the periplasm and activate proteases that convert the 20-kDa fragment of FecR to the 15-kDa fragment. Arguing against this possibility is our finding that FeCit that has experimentally accumulated in the periplasm does not induce transcription of the *fec* transport gene. In a more plausible model, FecA loaded with FeCit imposes a structural change in FecR that converts the 20-kDa fragment into a protease-sensitive form.

Not only RseP but also the periplasmic protease Prc is essential for FecR fragmentation (Braun and Hantke, unpublished results). *E. coli* K-12 BW25113 *fecB-lacZ* synthesizes β-galactosidase in response to FeCit in the medium. In a *prc* mutant of BW25113 obtained from the Keio collection and possessing Prc-typical conditional growth defects (Hara *et al*. 1991) β-galactosidase was not synthesized under growth-permitting conditions but its synthesis was restored following conversion of the *prc* mutant to *prc* wild-type. Without cleavage by Prc no FecR fragment can be formed by the RseP protease that stimulates FecI. The substrates of Prc remain to be determined.

## Regulated intramembrane proteolysis (RIP)

The proteolytic cascade initiated by OM receptors loaded with iron carriers ends in the cytoplasm with the formation of the N-terminal sigma-activating fragment (pro-sigma) or complete degradation (anti-sigma) of the sigma-regulatory proteins. Signal input occurs at the C-terminal end and signal output at the N-terminal end of the sigma regulatory proteins. The presence of the signal, manifested by the altered structure of the receptor, is conveyed to the regulatory proteins and presumably renders it sensitive to the proteases Prc, located in the periplasm, and RseP, located in the cytoplasmic membrane. The vectorial nature of the system, including the location of the regulatory proteins and the sequential fragmentation of the regulatory protein enable signal transfer from the OM receptor to the sigma factor in the cytoplasm. Regulated intramembrane proteolysis (RIP) is a feature that is widespread among prokaryotes and eukaryotes and allows them to communicate and transfer information between their environment and cellular compartments (Urban 2009). Among the processes regulated by RIP in prokaryotes are the iron supply, the stress response, cell division, sporulation, conjugation, cell polarity, and toxin expression. RIP involves the excision of a soluble protein fragment from an integral membrane protein that then acts as a signaling messenger. Transcription regulation elicited by iron carriers closely resembles that of genes that mediate the well-studied stress response of *E. coli* (Brooks and Buchanan 2008). In the stress response the exposed C-terminal ends of unfolded proteins in the periplasm elicit a stress signal recognized by the DegS protease. The latter cleaves the periplasmic domain from the RseA anti-sigma factor, which is then able to inhibit the formation of the σ^E^-RNA polymerase complex. In the next step, the RseP protease further cuts RseA within the membrane, close to the cytosolic side (Kanehara *et al*. 2002). Fragmented liberated RseA continues to inhibit σ^E^ until it is completely degraded by cytoplasmic ATP proteases.

RseP is part of the S2P family of Zn metalloproteases, which also includes mjS2P from *Methanocaldococcus jannaschii*. Determination of the X-ray structure of mjS2P (Feng *et al*. 2007) has provided insights into how a hydrophilic peptide bond is cleaved within a hydrophobic membrane. The protease exhibits two distinct conformations in the crystal. In the closed conformation, the active site is surrounded by transmembrane helices and is impermeable to substrate peptide. Water molecules gain access to zinc in a polar channel that opens to the cytosolic side. In the open conformation, two transmembrane helices separate from each other, exposing the active site to substrate entry. RseP-mediated degradation of the membrane-bound pro/anti-sigma fragments implied in the transcription regulation of genes encoding iron carriers involves a similar sequence of events (Akiyama *et al*. 2004).

## Transcriptional regulation of iron transport genes by Extracytoplasmic Sigma Factors (ECFs)

The transcription of genes encoding Fe^3+^siderophore synthesis and transport proteins in gram-negative bacteria is usually controlled by ECFs sigma factors (Helmann 2019, Casas-Pastor *et al*. 2021, Otero-Asman *et al*. 2019, Visca *et al*. 2002). Our early studies on the regulation of FeCit transport proteins contributed to the discovery of ECFs. After the sequence of *fecI* had been determined it was considered to be a transcription factor required for *fec* gene expression (Van Hove *et al*. 1990). Four years later a bioinformatics analysis showed that FecI and seven other proteins constituted a new class of sigma factors (Lonetto *et al*. 1994). Sigma factors initiate gene transcription by recruiting RNA polymerases to gene promoters. ECFs, the largest and most diverse group of sigma factors, respond to extracellular signals that we previously referred to as “cell surface signaling” or “transmembrane signaling or transmembrane transcription control” systems (Ochs *et al*. 1995, 1996, Braun 1997, Braun *et al*. 2006). The term “cell surface signaling” has been adopted by other research groups studying iron-regulated ECF sigma factors (Bastiaansen *et al*. 2015b). ECF sigma factors belong to the σ^70^ family but lack most of the σ1 and σ3 regions of σ^70.^ (Helmann 2002). They bind via regions σ2 and σ4 to the -−10 and −35 promoter regions. In *E. coli*, FecI was the second ECF sigma factor, after the stress response σ^E^ factor, whose structure and function was determined (Brooks and Buchanan 2008) and it became the type protein in the ECF classification. FecI-like σ factors were originally classified as ECF05–ECF10 (Staron *et al*. 2009, Pinto and Mascher 2016) but a recent analysis identified many more ECF sigma factors, such that ECF05–ECF9 are now contained in group ECF243, the largest group of the new classification (Casas-Pastor *et al*. 2021). An average of ten distinct ECFsigma factors are found per bacterial genome, although *E. coli* K12 encodes only two, FecI and σ^E^ (Braun and Mahren 2005). In addition to the assignment of FecI to ECF sigma factors by sequence homology, sigma factor function was determined experimentally. Purified RNA polymerase core enzyme supplemented with purified FecI transcribed a 330–nucleotide fragment of the *fecA* promoter DNA. Neither FecI nor RNA polymerase core enzyme alone directed transcription from the *fecA* promoter. No specific transcript was obtained with RNA polymerase charged with σ^70^. The affinity of the FecI RNA polymerase for the *fecA* promoter was significantly higher than that of σ^70^ RNA polymerase. Band shift experiments demonstrated binding of FecI to *fecA* promoter DNA only in combination with RNA polymerase core enzyme ruling out the possibility that FecI acts as a transcription activator that directly binds to DNA. Mobility band shift of *fecA* promoter DNA caused by cell lysates required growth of cells in the presence of FeCit and expression of FecA, FecI and FecR (Angerer *et al*. 1995).

ECF sigma regulation has also been extensively studied in *Pseudomonas* (Llamas *et al*. 2014) Visca *et al*. 2002). A phylogenetic analysis revealed as many as 253 ECF sigma factors in 14 different *Pseudomonas* strains (Otero-Asman *et al*. 2019). Transcriptional regulation of siderophores in *Pseudomonas* largely resembles that of the *fec* genes but an important difference is that the regulatory proteins act mainly as anti-sigma factors, although pro-sigma activities have been identified as well (see chapter 4.3).

## *In vitro* *fec* gene transcription. fur repressor binding sites

**Figure 7.**
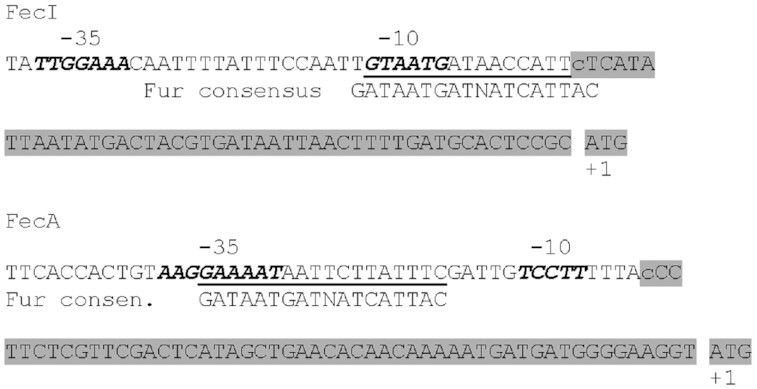
Promoter sequences of *fecI* and *fecA*. The transcribed sequences are highlighted in grey, transcription start sites shown in lower case, the -10 and -35 regions indicated in bold, Fur binding sites are underlined and compared with the Fur consensus sequence (Enz *et al*. 1995).

Both the *fecABCDE* genes and the *fecIR* genes are tandemly arranged, with a promoter upstream of *fecA* and *fecI*, respectively (Fig. 1). Transcription from either one yielded the mRNA fragments expected in response to FeCit-activated FecI and iron deficiency. Primer-extension experiments localized the transcription start sites for *fecA* and *fecI* (Fig. 7). The two promoters are regulated by the Fur repressor (Hantke 1981, Angerer and Braun 1998). Its binding site was localized in the −10 region of *fec*I and the -35 region of *fecA* (Fig. 7) (Enz *et al*. 1995). These sequences are poorly related to the −10 and −35 promoter consensus sequences of *E. coli* σ^70^-dependent promoters but homologous to promoters regulated by ECF σ factors (Lonetto *et al*. 1994).

Northern hybridization analysis demonstrated a 2.5-kb mRNA derived from the *fecA* gene and a 1.5-kb mRNA from the *fecIR* genes (Enz *et al*. 1995). Synthesis of the *fecA* mRNA was shown to be dependent on FeCit, FecIR, and a low iron concentration, established experimentally using the iron chelator dipyridyl. A polycistronic mRNA of *fecABCDE* was detected only by RT-PCR. The transcript starts at nucleotide -5 and expands over the hairpin structure located downstream of *fecA* that downregulates transcription of the poorly transcribed *fecBCDE* genes. A 6-kb mRNA fragment encodes *fecABCDE*, which is further trimmed to a more stable 2.5-kb *fecA* mRNA fragment. Synthesis of the 1.5-kb *fecIR* mRNA is regulated by Fur and not by FeCit through FecIR (Enz *et al*. 1995). Transcription of the *fecIR* genes is not autoregulated. Point mutations in the −10 region strongly reduce the level of *fecA* transcription and impair the binding of FecI-loaded RNA polymerase to *fecA* promoter DNA (Enz *et al*. 2003b) whereas point mutations in the −35 region have a much smaller effect on transcription. The strong reduction in transcription resulting from mutations downstream of the *fecA* transcription start site (Angerer *et al*. 1995, Enz *et al*. 2003b) suggests the involvement of the downstream region in a subsequent step of transcription activation, for example, conversion of the conformation of the RNA polymerase holoenzyme from closed to open.

Footprinting scans of the Mn^2+^-loaded Fur repressor (in which Fe^2+^ is replaced by more stable Mn^2+^) showed that it covers positions −38 to −1 of the coding strand of the *fecA* promoter, thus including the Fur consensus sequence at positions −36 to −17 (Fig. 7) (Enz *et al*. 1995). The levels of the resulting mRNA correspond to those of the encoded proteins, among which FecA is by far the most prominent. *fecA-lacZ* transcription is ten-fold higher than *fecI-lacZ* transcription. *In vitro* experiments with isolated proteins (Mahren and Braun 2003) showed binding of the FecI sigma factor to the β’ subunit and to the truncated β’ _1-313_ fragment of RNA polymerase and that FecR promotes binding.

## Transcription regulation of the outer membrane heme transport gene by an extracytoplasmic function sigma factor in *Serratia marcescens*

Quantitative studies of heme transport gene regulation, and specifically of extracellular signaling across the OM, have been performed in *Serratia marcescens*. Signaling involves ECF sigma factor regulation similar to gene regulation of FeCit transport and ferric siderophore transport. However, heme transport and its regulation also have unique characteristics. Heme is an abundant iron source that is acquired by many bacteria to meet their iron demand (Wandersman and Stojiljkovic 2000, Cescau *et al*. 2007). Heme may occur in free form but most of it is contained in proteins such as hemoglobin, myoglobin, and hemopexin. Consequently, the cellular uptake of heme requires its prior extraction from proteins. In *S. marcescens* and other gram-negative bacteria, this is achieved by the excretion of hemophores that scavenge exogenous heme and heme from hemeproteins in the extracellular medium. Hemophores have a very high affinity for heme, with a Kd value of the heme-hemophore complex in the picomolar range and thus similar to the Kd values of heme-globin complexes. Hemophores form a family of heme-binding proteins without homology to other known proteins. Fundamental studies on hemophore-mediated heme import have been performed with the heme transport system of *S. marcescens*, discussed herein.

Heme transport gene regulation has been studied by transferring genome fragments of *S. marcescens* into an *E .coli* mutant, which is incapable of heme uptake and heme synthesis. A clone that conferred growth encoded the HasR outer membrane receptor (Ghigo *et al*. 1997). Heme has a low solubility and a size of 612 Da. Both properties favor energized transport via OM receptors that mediate the transport of substrates at low concentrations and larger than 600 Da. The further transport of heme into the *S. marcescens* cytoplasm is achieved via a heme-specific ABC transporter (Létoffé *et al*. 2008), encoded by *hemTUV*, which is located close to the *has* operon. In *E. coli* transport of heme across the cytoplasmic membrane is mediated by a dipeptide permease (Létoffé *et al*. 2006).

**Figure 8.**
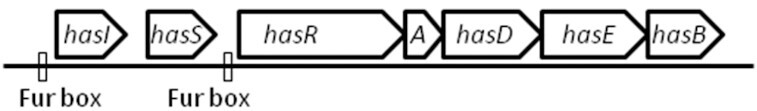
Arrangement of the *has* genes of *Serratia marcescens hasI* encodes the sigma factor, *hasS* the anti-sigma factor, *hasR* the OM transport and regulatory protein, *hasA* the hemophore, *hasD* and *hasE* the proteins for HasA secretion, *hasB* the HasR-specific TonB paralog. Fur denotes the binding sites of the iron-loaded Fur repressor (Rossi *et al*. 2003).

Only the *has* operon (Fig. 8) is regulated by an ECF sigma/anti-sigma mechanism. The operon starts with *hasI* and *hasS*, which encode a sigma factor and an anti-sigma factor, respectively. The two genes are followed by *hasR*, which encodes the HasR OM heme transport protein, *hasA*, the structural gene of the hemophore, *hasD* and *hasE*, which encode two hemophore secretion proteins active in the cytoplasmic membrane, and *hasB*, encoding a TonB homologue specific for heme uptake via HasR (Paquelin *et al*. 2001, Rossi *et al*. 2003, Létoffé *et al*. 2004). HasB is inactive in *E. coli*. In addition to heme transport across the OM, HasR initiates *hasR* transcription by the HasA hemophore together with heme. The structure and function of HasR are similar to that of FecA in *E. coli* and FpvA in *P. aeruginosa*. The protein consists of a β-barrel with a plug inside that forms a heme-specific channel (Létoffé *et al*. 2005). This specificity is a particular property of HasR, since pores of the other OM transporters display no substrate specificity. Substrates are specifically recognized by surface-exposed residues. In addition, HasR contains an N-terminal extension required for the initiation of *hasR* transcription induced by external transport substrates.

Transcription initiation was studied by investigating the interaction of HasR with heme-loaded and unloaded (free) HasA and the transfer of heme from HasA to HasR (Wandersman and Delepelaire 2012). The high-affinity binding of the heme-loaded or free hemophore to the receptor and the subsequent transfer of heme from the hemophore to the receptor are energy-independent reactions (Cescau *et al*. 2007). In cells expressing the OM transporter HasR, the dissociation constant for heme-loaded and free hemophore is the same, 5 nM (Létoffé *et al*. 1999, 2000, 2003). Free hemophore assumes the same overall structure as the heme-loaded form but the loop carrying the H32 iron ligand is widely displaced. However, contrary to expectation, it is not heme-loaded hemophore that initiates *hasR* transcription. In a HasA triple mutant protein that does not bind heme but still binds to the HasR receptor with high affinity, the *has* signaling cascade is fully induced in response to heme (Létoffé *et al*. 2004; Cescau *et al*. 2007). Binding of heme to the hemophore is not required for induction; rather, induction is elicited by heme adsorption to HasR and the presence of the unloaded hemophore. Since heme is transported into the cell but the hemophore remains outside, there is a clear distinction between transport and signaling. Creating this distinction in the FeCit and Fe-siderophore transport systems required the isolation of mutants in the OM receptor gene that maintained signaling but had lost transport.

Heme is transferred *in vitro* from its high-affinity site on HasA to its low-affinity site in HasR against a 10^5^ affinity gradient. Structural changes occur in both proteins. After heme transfer from HasA to HasR, free HasA remains bound to HasR. Dissociation of the free hemophore from the receptor consumes energy which is actually the only energy-requiring reaction during heme translocation across the OM. In the system reconstituted in *E*. *coli*, the enhanced energy demand is provided through a 15-fold stronger expression of the Ton system (TonB-ExbB-ExbD) by plasmid-encoded *tonB exbB exbD* genes (Létoffé *et al*. 2004). Energy-driven conformational changes in the HasR plug domain to which TonB binds (Ratliff *et al*. 2021) might rearrange the HasR extracellular loops, allowing free hemophore release. The uptake of free heme does not require *tonB exbB exbD* gene overexpression.

### *has* gene regulation deviates from *E. coli fec* gene and Pseudomonas Fe^3+^siderophore transport gene regulation

Heme regulatory genes, their arrangement, and the functions they encode are similar to the FeCit system (compare Fig. 8 with Fig. 1). The HasI protein shows 30.3% sequence identity to the *E. coli* FecI protein, and the HasS protein 23.9% sequence identity to the *E.coli* FecR protein. The high structural similarity of the HasIS proteins to the functionally known FecIR proteins suggests a sigma function of HasI and an anti-sigma function of HasS. Mutants in *hasA, hasI*, or *hasR* no longer multiply in iron-extracted medium adjusted to a low hemoglobin concentration of 0.5 µM (Rossi *et al*. 2003). Wild-type cells produce HasR and HasA in iron chelated medium in contrast to *a hasI::km* mutant that is devoid of HasR and HasI. This result is supported by the use of a transcriptional *lacZ* fusion to the *hasR* promoter, which exhibits high β-galactosidase activity in the *hasI+* wild-type but only residual levels in the *hasI^–^* mutant. Sigma factors regulating iron uptake systems do not activate transcription of their own gene nor of the gene of the related anti-sigma factor. In contrast, HasI regulates *hasS* gene expression (Biville *et al*. 2004). Cells expressing *hasS* and *hasI* have only basal levels of HasS, consistent with an anti-sigma activity of HasS on *hasI* transcription. Introduction of a plasmid encoding HasS strongly reduces the β-galactosidase levels, indicating negative *hasR* regulation by HasS. HasR occupied with heme-loaded hemophore inhibits the anti-sigma activity of HasS. HasI induces the transcription of *hasS* which results in the accumulation of inactive HasS. Under conditions of heme shortage HasR no longer signals and HasS becomes fully active and turns off transcription. Heme loaded hemophore induces *hasS* transcription but not *hasI* transcription and inhibits anti-sigma activity of HasS. Only the anti-sigma gene is positively regulated by the sigma factor. The nucleotide sequence TTTACGGGTTT is contained in the -35 region of *hasS* and *hasR* and may be the HasI-specific promoter target sequence. Site-directed mutagenesis of the putative HasI binding site upstream of *hasS* demonstrates that this sequence is required for HasI-mediated *hasS* transcription. Why should *hasS* transcription be regulated by HasI? During the heme shortage, the rapid termination of *hasR* transcription by the accumulated HasS avoids the wasteful synthesis of the highly expressed HasR protein. *hasI* but not *hasS* is subject to Fe-Fur repression and the expression of HasI in a *fur* mutant increases *hasS* expression by 40-fold. *hasI* is not autoregulated.

Analogous to ferric citrate and Fe^3+^siderophore transport gene transcription initiation HasS was supposed to interact with the periplasmic signaling domain of HasR. A fragment of HasS consisting of 78 C-terminal residues was isolated and its structure determined (Malki *et al*. 2014). The fragment is soluble only in the presence of a detergent, and adopts a monomeric elongated form with distinct secondary structure elements but lacks a stable tertiary structure. Its sequence suggests a high degree of differential mobility. Partial unfolding is consistent with the protease sensitivity of FecR and the Pseudomonas regulatory proteins. The elongated shape allows the fragment to span the width of the periplasm.

The solution structure of the periplasmic signaling domain of HasR includes 21 additional residues containing the TonB/HasB box (Malki *et al*. 2014). The signaling domain (residues 8–90) forms a globular fold to which an unstructured flexible region is bound that represents the adjacent TonB/HasB box. Addition of the signaling domain to the HasS fragment quenches the intrinsic fluorescence of HasS, associated with a change in the environment of the tryptophan side chains. Heteronuclear NMR analysis revealed a reduction of the overall intensity of the HasS fragment spectrum by nearly 35% in the presence of the signaling domain. Two regions in that domain are involved in the interaction with HasS: one is present on the protein surface and highly accessible and the other is located in a groove between two helices. The HasR/HasS interacting region corresponds to the PupB/PupR structure (Jensen *et al*. 2020) and to the location of mutations in the FecA signaling domain that severely affect signaling (Breidenstein *et al*. 2006). In the presence of the fragment and under iron restricted conditions, the level of heme-induced synthesis of HasR and HasA is much lower than that in cells lacking the HasS fragment. The HasS fragment interferes with the transcription-inducing signaling cascade. Understanding the mechanism of *hasR hasS* transcription initiation by free heme and heme bound to hemophore awaits elucidation of the mechanisms underlying signal transfer by HasS across the periplasm into the cytoplasm and the interaction of HasS with HasI. Presumably, signal transfer involves regulated proteolysis of HasS for which the proteases and the proteolytic fragments should be identified.

Searches of the gene bank reveal the heme-induced transcription activation of heme transport genes of the *S. marcescens* Has type in other gram-negative bacteria such as strains of *Pseudomonas*, *Yersinia*, *Klebsiella*, *Bordetella*, *Xanthomonas*, *Porphyromonas*, *Pectobacteria*, and *Halomonas* (Huang and Wilks 2017). All have been experimentally studied to a much smaller extent than *S. marcescens*.

## Summary

Transcription regulation of the genes encoding the *E. coli* Fe^3+^citrate transport, the Pseudomonas Fe^3+^siderophore transport and synthesis and the *S. marcescens* heme outer membrane protein share a number of extraordinary features:

### Transcription is initiated by extracellular iron carriers

The presence of the iron carriers at the cell surface is recognized by receptor proteins in the OM.

OM proteins exert a dual function: (i) recognition of the iron carriers and the subsequent transfer of the resulting signal across the OM into the periplasm; (ii) transport of the iron carriers into the periplasm.

Energy provided by the proton-motive force of the cytoplasmic membrane is required for both signaling and transport activities and is transferred to OM proteins by the TonB ExbB ExbD protein complex.

Binding of the iron carriers to their receptors initiates conformational changes in the receptors that extend from the cell surface to the signaling domain in the periplasm.

The signaling domains interact with the C-proximal region of the sigma regulatory proteins in the periplasm.

The regulatory proteins extend from the OM, through the periplasm across the cytoplasmic membrane into the cytoplasm.

Signal input takes place at the C-terminus and signal output at the N-terminus of the regulatory proteins.

Transmission of the signal from the C-terminus to the N-terminus is achieved by a directed proteolytic cascade.

An N-terminal precursor fragment of 20-kDa is excised from the regulatory proteins by a chemical self-cleaving process at a Gly-Thr bond.

The regulatory proteins are presumably sequentially fragmented, from 20 kDa to 15 kDa to 12 kDa, by the Prc protease and possibly additional proteases and finally by the RseP protease. The resulting N-terminal fragment in the cytoplasm either binds to and activates sigma proteins (pro-sigma activity) or is released from the sigma proteins by proteolysis (anti-sigma activity).

The sigma-activating fragment of a regulatory protein contains a highly conserved N-proximal sequence of ∼50 residues that binds to σ_4_ of sigma factors.

The cellular iron level is regulated via the rapid repression of regulatory genes, transport genes, and synthetase genes by the iron-loaded Fur protein and further adapted through a slower process by ECF sigma factor-mediated transcription initiation through extracellular ferric citrate, Fe^3+^siderophores or heme.

## Outlook

The complex pathways that regulate iron transport offer sites of interference with the growth of pathogenic species, which in their natural habitats are frequently exposed to iron-limiting conditions. The current model of the transcription activation of Fe^3+^siderophore transport genes is mainly based on molecular genetic experiments. Studies that make use of biochemical and biophysical techniques, as applied to investigations of heme transport, should also be employed in the other systems. To fully understand the steps involved in the signaling cascade from the cell surface to the cytoplasm, several questions must be answered: What is the molecular basis of receptor energization by TonB-ExbB-ExbD? How does the TonB-ExbB-ExbD complex react to the proton-motive force and how does TonB transfer energy to the receptors? Does TonB energize signaling via the same mechanism that it uses to energize transport? How do the structural changes in the receptors that occur during transport differ from those that occur during signaling?

Studies with purified proteins will reveal the physical mechanism underlying the conversion of regulatory proteins to protease-sensitive proteins. Determination of the complete set of periplasmic and cytoplasmic proteases and their substrates will contribute to the development of a realistic model of the regulated proteolysis pathway. The proteolytic fragmentation of regulatory proteins to sigma-active products can be followed by mass spectroscopy, which thus offers a novel approach to elucidating the molecular reactions that drive transcription initiation. Such studies would disclose the bypass reactions that avoid the formation of the 20-kDa fragment in wild-type cells devoid of the Gly-Thr sequence and in mutants of cloned genes. The mechanism of sigma activation by pro-sigma and anti-sigma factors is another field that merits further exploration. This can perhaps be best accomplished in the Fec system of *E. coli* K-12, as the system components are relatively simple and easy to manipulate genetically and the bacterium does not synthesize interfering siderophores of the Fec type.
